# Unraveling the transcriptome-based network of tfh cells in primary sjogren syndrome: insights from a systems biology approach

**DOI:** 10.3389/fimmu.2023.1216379

**Published:** 2023-08-10

**Authors:** Danyang Luo, Lei Li, Yi Yang, Yulin Ye, Jiawei Hu, Yuan Zong, Jiawen Zhao, Yiming Gao, Haimin Xu, Ning Li, Yinyin Xie, Liting Jiang

**Affiliations:** ^1^ Department of Stomatology, Ruijin Hospital, Shanghai Jiao Tong University School of Medicine, Shanghai, China; ^2^ College of Stomatology, Shanghai Jiao Tong University, Shanghai, China; ^3^ Department of Pathology, Ruijin Hospital, Shanghai Jiao Tong University School of Medicine, Shanghai, China; ^4^ Shanghai Institute of Hematology, State Key Laboratory of Medical Genomics, National Research Center for Translational Medicine at Shanghai, Shanghai, China; ^5^ Ruijin Hospital, Shanghai Jiao Tong University School of Medicine, Shanghai, China

**Keywords:** primary Sjogren’s syndrome, labial salivary gland, T follicular helper cell, transcriptome sequencing, interferon

## Abstract

**Background:**

Primary Sjogren Syndrome (pSS) is an autoimmune disease characterized by immune cell infiltration. While the presence of follicular T helper (Tfh) cells in the glandular microenvironment has been observed, their biological functions and clinical significance remain poorly understood.

**Methods:**

We enrolled a total of 106 patients with pSS and 46 patients without pSS for this study. Clinical data and labial salivary gland (LSG) biopsies were collected from all participants. Histological staining was performed to assess the distribution of Tfh cells and B cells. Transcriptome analysis using RNA-sequencing (RNA-seq) was conducted on 56 patients with pSS and 26 patients without pSS to uncover the underlying molecular mechanisms of Tfh cells. To categorize patients, we employed the single-sample gene set enrichment analysis (ssGSEA) algorithm, dividing them into low- and high-Tfh groups. We then utilized gene set enrichment analysis (GSEA), weighted gene co-expression network analysis (WGCNA), and deconvolution tools to explore functional and immune infiltration differences between the low- and high-Tfh groups.

**Results:**

Patients with pSS had a higher positive rate of the antinuclear antibody (ANA), anti-Ro52, anti-SSA, anti-SSB and hypergammaglobulinaemia and higher levels of serum IgG compared to the non-pSS. Histopathologic analyses revealed the presence of Tfh cells (CD4^+^CXCR5^+^ICOS^+^) in germinal centers (GC) within the labial glands of pSS patients. GSEA, WGCNA, and correlation analysis indicated that the high-Tfh group was associated with an immune response related to virus-mediated IFN response and metabolic processes, primarily characterized by hypoxia, elevated glycolysis, and oxidative phosphorylation levels. In pSS, most immune cell types exhibited significantly higher infiltration levels in the high-Tfh group compared to the low-Tfh group. Additionally, patients in the Tfh-high group demonstrated a higher positive rate of the ANA, rheumatoid factor (RF), and hypergammaglobulinaemia, as well as higher serum IgG levels.

**Conclusion:**

Our study suggests that Tfh cells may play a crucial role in the pathogenesis of pSS and could serve as potential therapeutic targets in pSS patients.

## Introduction

Primary Sjogren’s Syndrome (pSS) is an autoimmune disease characterized by persistent lymphocyte infiltration in exocrine glands, including salivary and lacrimal glands, leading to Sicca syndrome (1). The prevalence ratio of females to males was 10.72 and the overall age of pSS patients was 56.16 years (2). Similar to other rheumatic autoimmune diseases, the pathogenesis of pSS is considered multifactorial, involving genetic susceptibility and environmental factors, contributing to autoimmunity and chronic inflammation (3). The disease manifests with a wide range of glandular and extraglandular symptoms, with more than 80% of pSS patients experiencing Sicca syndrome, fatigue, and joint pain (4). Infiltration of the labial salivary gland (LSG) by T cells, B cells, plasma cells, and macrophages, akin to other salivary glands and lacrimal glands, is a prominent pathological feature of pSS (5). Notably, increased T-cell infiltration in exocrine glands correlates significantly with inflammation, detrimental characteristics, and disease progression (6).

T cells typically exit secondary lymphoid organs in search of infected cells throughout the body. Their effector functions and ability to combat infections rely on direct interactions with antigen-presenting cells (APCs) (7). However, dysfunctional T-cell regulatory pathways contribute to the development of autoimmune disease (8). In pSS, T cells are activated by various triggers, such as virus infections or even physical desiccation, while the immune response of glandular epithelial cells further amplifies T cell activation (9). Among T cells, CD4^+^ T helper (Th) cells have been implicated in positively influencing B cells to produce antibodies against SSA antigens in pSS patients (10). Advances in research have led to the identification of various Th cell subsets, including Th0, Th1, Th2, Th17, regulatory T (Treg), T follicular helper (Tfh) cells, and T follicular regulatory (Tfr) cells, based on distinct cytokine patterns and transcription factors (11). Despite the recognized importance of Th cells in pSS, the underlying mechanisms of their action remain elusive.

Tfh cells have emerged as a distinct Th lineage specialized in supporting B cell maturation and survival during germinal centers (GC) in secondary lymphoid tissues (12, 13). The immunophenotype of Tfh cells is characterized by sustained surface expression of CXCR5, ICOS, and PD-1, intracellular expression of crucial transcription factors Bcl-6 and Ascl-2, and abundant secretion of IL-21 (14, 15). Tfh cell differentiation is a multistage, multifactorial process, involving interactions between IL-6, ICOS, IL-2, and TCR signaling during dendritic cell (DC) priming, which regulates the expression of CXCR5 and Bcl6. Tfh cells interact with antigen-specific B cells in the follicle, interfollicular zone, or the T-B border, and play a role in GC (16). However, in recent years, various research groups have focused on exploring the relationship between pSS and ectopic GC, which exhibit inconsistencies in detection methods, leading to variations in their reported occurrence (17, 18). In pSS patients with GC, upregulation of salivary gland CXCR5, IL-21, and the ICOS co-stimulatory pathway has been observed. Additionally, the peripheral blood of pSS patients with germinal centers shows a significant increase in CD4^+^CXCR5^+^PD-1^+^ICOS^+^ Tfh cells, which are the main producers of IL-21 and are closely associated with increased circulating IgG levels and decreased complement C4. Furthermore, within the salivary gland tissue, CD4^+^CD45RO^+^ICOS^+^PD1^+^ cells selectively infiltrate the ectopic lymphoid tissue (19). However, there is limited research on the distribution and expression of Tfh cells across different degrees of pSS. By integrating our RNA sequencing data with pSS datasets from the NCBI Gene Expression Omnibus (GEO) database, we identified seven hub genes that exhibited significantly elevated expression in the higher focus score (FS) group, and all of these genes were strongly positively correlated with Tfh cell infiltration (20).

To further investigate the association between Tfh cells and pSS, this study aims to examine the distribution and gene expression profile of Tfh cells in pSS patients through the integration of histopathology and transcriptome analysis. Our findings indicate that a subset of pSS patients displays the elevated expression of genes associated with Tfh cells. Based on the degree of Tfh cell infiltration, we categorized pSS patients into Tfh-high and Tfh-low groups, revealing that the Tfh-high group exhibited an enhanced inflammatory response, altered metabolic process, and glandular immune microenvironment. This study provides novel insights into the role of Tfh cells in the progression of pSS.

## Materials and methods

### Participants’ information and sample collection

A total of 152 patients who underwent labial salivary gland (LSG) biopsy at the Department of Stomatology, Ruijin Hospital, Shanghai Jiaotong University School of Medicine, due to Sicca syndrome or abnormal laboratory indicators, were included in this study. Based on the 2016 American College of Rheumatology/European League Against Rheumatism (ACR/EULAR) classification principles (21), the study enrolled 106 patients with pSS, with a mean age of 49.26, and 46 patients with non-pSS, with a mean age of 52.48. The female patients accounted for 87.7% and 80.4% of the respective groups. The non-pSS group consisted of patients with sicca symptoms who did not meet the classification criteria for pSS. Detailed clinical data can be found in **Table 1**; **Supplementary Table S1**. Before the biopsy, none of the patients had received any medication, such as immunosuppressive or steroid agents, orally or otherwise, that could result in dryness of the mouth. All patients provided written informed consent before the collection of clinical information and LSG samples after the biopsy. The Ethics Committee of Ruijin Hospital, Shanghai Jiao Tong University School of Medicine, and the Chinese Clinical Trial Registry (ChiCTR2000039820) reviewed and approved this study.

**Table 1:** Phenotypes of pSS and non-pSS patients compared according to 2016 ACR/EULAR diagnostic criteria.

Clinical characteristic	pSS (n=106)	Non-pSS (n=46)
*Gender (Female)*	93/106	37/46
*Age*	48.50 ± 12.19	52.48 ± 12.23
*ANA+ (%)*	91.5**	71.7
*Anti-SSA+ (%)*	87.7***	26.1
*Anti-Ro52+(%)*	72.6**	45.7
*Anti-SSB+ (%)*	34.0***	6.5
*IgG (g/L)*	18.21 ± 9.10***	12.74 ± 3.79
*IgA (g/L)*	2.90 ± 1.30	2.68 ± 1.03
*IgM (g/L)*	1.50 ± 0.88	1.47 ± 0.82
*C3 (g/L)*	1.11 ± 0.23	1.13 ± 0.19
*C4 (g/L)*	0.27 ± 0.09	0.31 ± 0.12
*Hypergammaglobulinaemia (%)*	28.3**	10.9
*Hypocomplementemia (%)*	2.8	6.5

Data are mean ± standard deviation (SD) or n (%). *p<0.05; **p<0.01; ***p<0.001.

ANA, anti-nucleic antibody.

### Histological staining

Hematoxylin and eosin (HE) staining, immunohistochemistry (IHC) staining, and immunofluorescence (IF) staining were performed using LSG tissues, following the manufacturer’s instructions. Briefly, the LSG samples were first fixed in fresh 4% neutral formaldehyde overnight. Subsequently, they were automatically dehydrated using an automatic tissue dehydrator, embedded in paraffin, and cut into 5-mm-thick serial sections for staining. For HE staining, the sections were rehydrated and stained with Mayer’s hematoxylin for 6 min and eosin for 10 seconds. For IHC staining, LSG tissue sections were degummed and rehydrated in graded ethanol for blocking and antigen retrieval. This was followed by incubation with primary antibodies (see **Supplementary Table S2**) at 25°C for 20 minutes. Sections were washed with PBS, incubated with a secondary antibody, stained with 3,3’-diaminobenzidine (DAB, K5007, DAKO, Denmark), and counterstained with hematoxylin. Slices were then visualized using the BOND Polymer Refine Detection Kit (DS9800, Leica Biosystems) on a Leica Bond RX automated staining platform (Leica Biosystems). Histological images were captured using a microscope (Nikon Eclipse Ni-U) equipped with a Nikon DS-Ri digital camera. For co-localization purposes, IF staining for CD4/CXCR5 was performed on rehydrated sections. The staining was amplified using the tyramide signal amplification technique (TSA Plus Fluorescein Kit, Runnerbio, China) according to the manufacturer’s protocol. Fluorescence images were captured using a Leica TCS SP8 MP confocal microscope (Leica, Wetzlar, Germany).

### RNA sequencing data and basic analysis

RNA sequencing was conducted using LSG tissues obtained from a total of 82 patients, comprising 56 patients with pSS and 26 patients with non-pSS. Total RNA was extracted from each patient using TRIzol Reagent (Invitrogen, USA) and quantified through various methods, including the Agilent 2100 bioanalyzer (Agilent Technologies, CA, USA), NanoDrop (Thermo Fisher Scientific Inc.), and 1% agarose gel after verification. The library construction followed the manufacturer’s protocol, utilizing 1µg of total RNA. The library was then multiplexed and loaded into the Illumina Novaseq instrument (Illumina, CA, USA) for sequencing, employing a 2 × 150 bp paired-end (PE) configuration. The HiSeq (HCS), OLB, and pipeline -1.6 (Illumina) management software were employed to analyze and identify the basic HiSeq camera. Cutadapt (V1.9.1) was used to process the fastq data and filter it to obtain clean, high-quality data.

DESeq2 Bioconductor was used for differential expression genes (DEGs) analysis, with genes having a Padj < 0.05 being considered significantly different expression levels. Venn diagrams and volcano diagrams were generated based on the resulting data. The STRING database (http://string-db.org) (22) was utilized to assess interaction relationships among overlapping genes and construct protein-protein interaction (PPI) networks. GO functional annotation and Kyoto Encyclopedia of Genes and Genomes (KEGG) pathway enrichment analyses were conducted using the cluster profiler package in R (23). Additionally, Metascape analyses (24) were performed to identify ontology terms based on significant commonalities among genes in GO and KEGG. For gene set enrichment analysis (GSEA), the normalized data were analyzed using the GSEA software tool (version 4.3.2) (https://www.gsea-msigdb.org/gsea/index.jsp) (25, 26). The correlation between the Tfh marker genes and sereval interested pathway (Oxidative phosphorylation, glycolysis, hypoxia and epithelial mescenchymal transition) genes was calculated using Spearman’s rank correlation. Results were visualized using the R package ggplot2.

### Weighted gene co-expression network analysis

Weighted Gene Co-expression Network Analysis (WGCNA) was performed to identify genes most relevant to Tfh cell infiltration using the WGCNA R package (27, 28). Among the 82 samples, only genes with Fragments Per Kilobase Million (FPKM) values > 0.3 were included in the analysis. All differentially expressed genes identified were used to generate gene co-expression modules. The association between module eigenvalues, Tfh cell infiltration groups, and clinical indicators was evaluated using the Wilcoxon rank sum test.

### Tfh cell infiltration and the single-sample gene set enrichment analysis

The cell type of each cluster, comprising 43 immune cells and 7 salivary gland components (**Supplementary Table S3**), was determined using the CellMarker 2.0 database (http://bio-bigdata.hrbmu.edu.cn/CellMarker/) by matching marker genes with known signature genes reported in previous studies (29). The proportion of immune cell types in the labial salivary glands (LSG) of patients with pSS was estimated using the single-sample gene set enrichment analysis (ssGSEA) (25) and the CIBERSORT (http://cibersort.stanford.edu) algorithms. Based on the degree of Tfh cell infiltration, patients with pSS were divided into two groups: the High Tfh cell infiltration group and the low Tfh cell infiltration group. Stromal scores, Immune scores, and ESTIMATE scores were calculated for LSG samples from different Tfh cell infiltration groups using the ESTIMATE R software package. The R packages ggplor2, ggpubr, and ggExtra were used for graphical visulizaiton. The correlation analysis of immune cells and glandular related genes was visualized by the “Corrplot” R package (version 0.92).

### Statistical analysis

Statistical tests, including independent sample t-tests, were conducted using GraphPad (GraphPad Software, San Diego, California USA, www.graphpad.com). Qualitative data were presented as percentages (%), and quantitative data were expressed as mean ± standard deviation (mean ± SD). A p-value of < 0.05 (*p<0.05; **p<0.01; ***p<0.001) was considered statistically significant difference.

## Results

### Characteristics of study subjects

**Figure 1:**
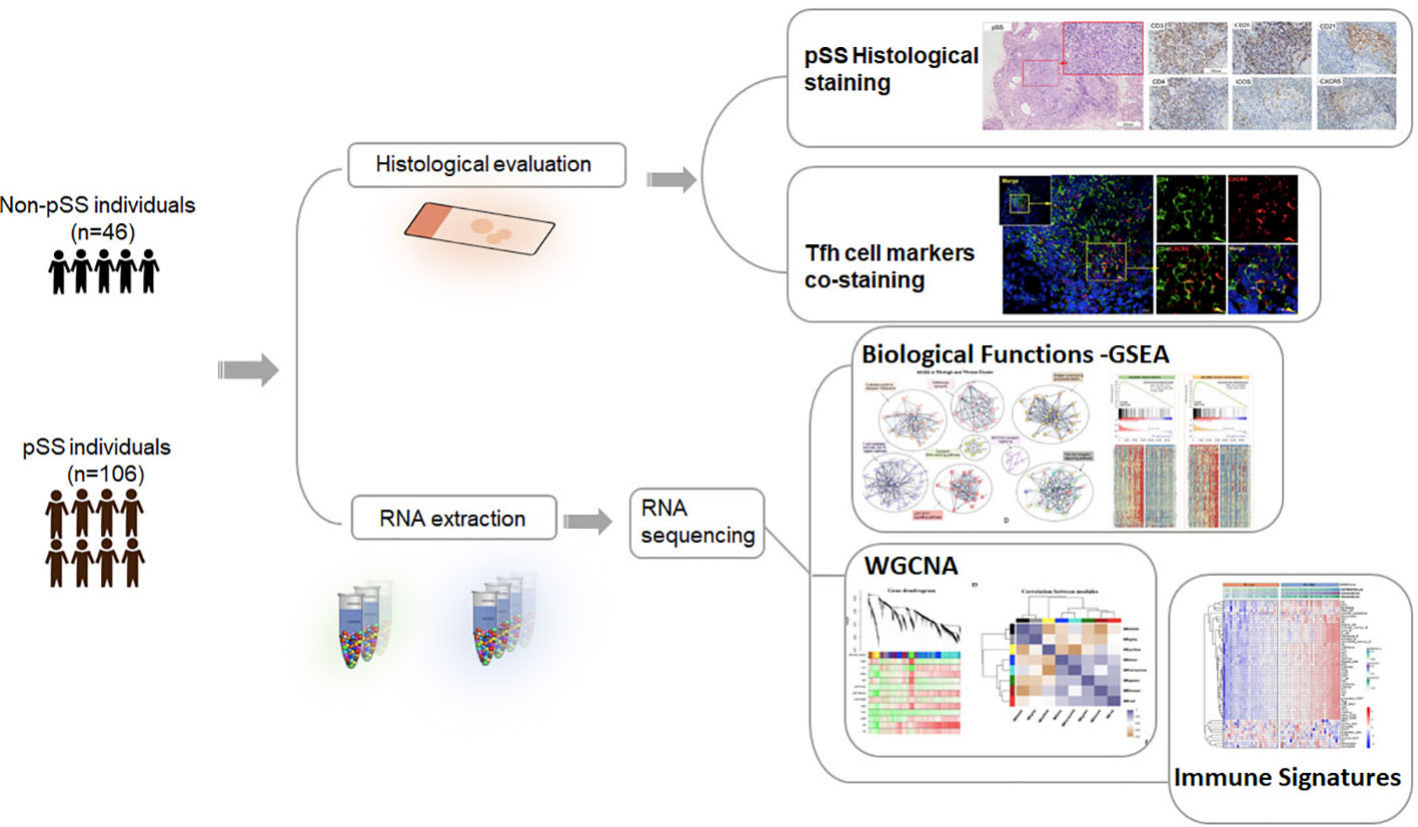
Schematic diagram of the workflow for the analysis of the histology and transcriptome.

A total of 152 patients were included in this study. The methodology and important results are summarized in **Figure 1**. **Table 1** provides an overview of the key characteristics of the subjects. There were no significant differences in gender and age between the pSS and non-pSS groups. However, patients in the pSS group exhibited a higher positive rate of the antinuclear antibody (ANA), anti-Ro autoantibodies 52 (anti-Ro52), anti-Sjögren syndrome antigen A and B (anti-SSA and anti-SSB) compared to those of the non-pSS group. Additionally, patients with pSS had higher levels of serum IgG (p<0.001) and a higher positive rate of hypergammaglobulinaemia (p < 0.01) compared to the non-pSS group.

### Different distribution of Tfh cells in the LSG of pSS patients

**Figure 2:**
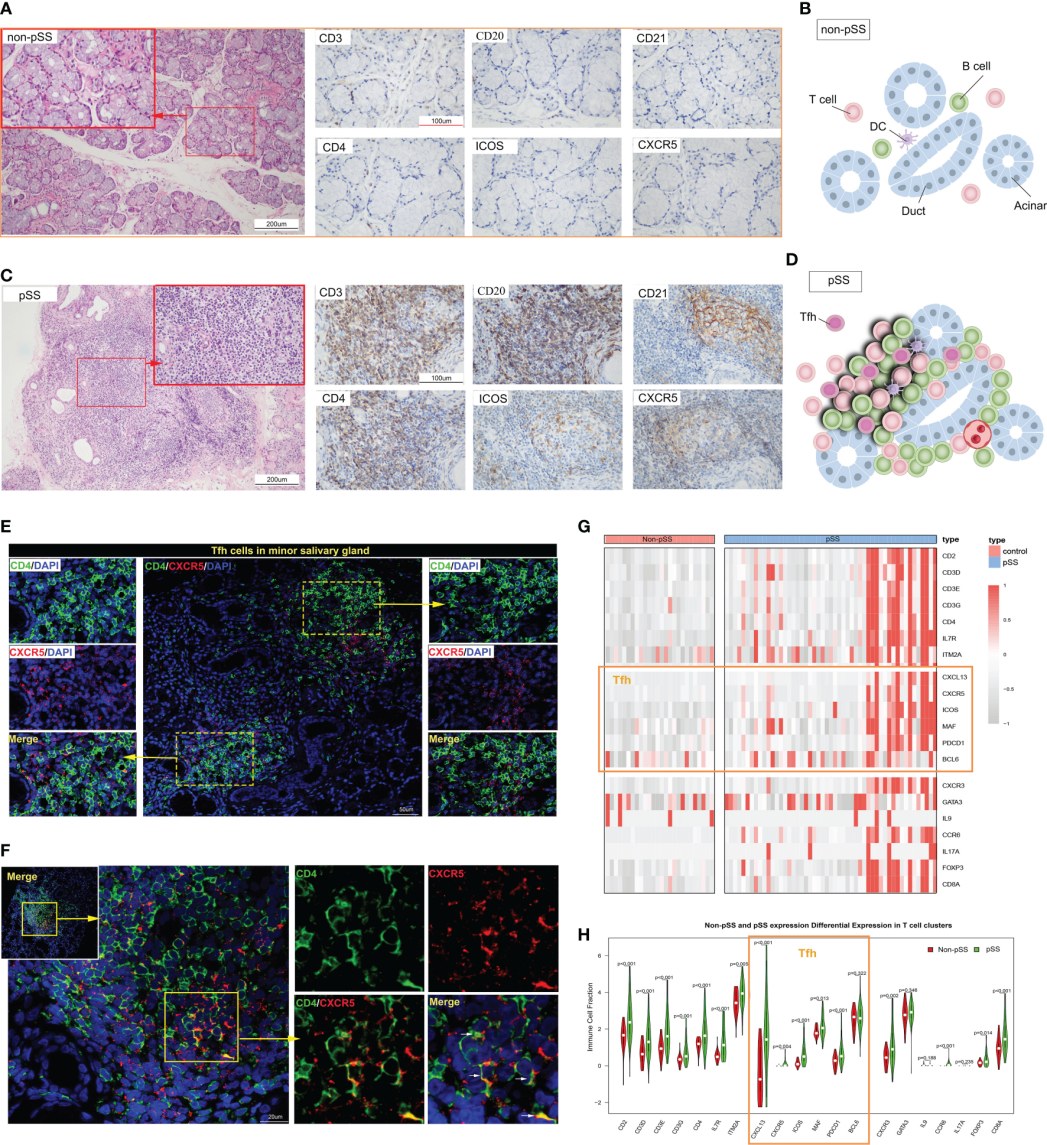
The distribution of follicular T helper (Tfh) cells in the immune microenvironment of labial salivary glands (LSGs) of patients with primary Sjogren’s syndrome (pSS). **(A)** Histological staining of LSGs in non-pSS patients showed that the left Hematoxylin and eosin (HE) staining showed that the gland structure was intact, and the right immunohistochemical (IHC) staining showed no or very small amount of lymphocytes infiltration (HE stain, original magnification ×200 or ×400; CD3, CD20, CD21, CD4, ICOS and CXCR5 IHC stain, original magnification ×400). **(B)** Schematic representation of salivary gland structure and the distribution of immune cells in non-pSS patients. **(C)** Serial sections of LSGs from representative patients with pSS, including HE and IHC staining. HE staining suggested the presence of a germinal centre (GC)-like structure in the glandular tissue. The presence of a GC-like structure was confirmed by IHC staining for CD3, CD20, and CD21. CD4, ICOS and CXCR5 IHC staining could distinguish the distribution of Tfh cells (HE stain, original magnification ×200 or ×400; IHC stain, original magnification ×400). **(D)** Schematic representation of salivary gland GC-like structure in patients with pSS. **(E, F)** Immunofluorescence (IF) co-staining of CD4, CXCR5 and DAPI in the smaller lesions **(E)** and larger lesions **(F)** of LSGs in patients with pSS. **(G)** Heat map represents the expression of the marker genes of the subsets of T cells in non-pSS and pSS patients. **(H)** The violin diagram depicted the differential expression of marker genes of T cell subsets in patients with pSS and non-pSS.

In addition to the activation of innate immunity and abnormal B cell activation, T cell activation also plays a crucial role in the pathogenesis of pSS. CD3^+^T cells are important components of the focal lesion. Moreover, according to a previous study (20), a special subset of CD4^+^T cells, Tfh cells, play an important role in pSS. We first utilized histology to observe the distribution of T cells, B cells, and follicular DC (FDC), particularly CD4^+^T and Tfh cells. In the LSG of non-pSS patients, CD21 expression was minimal or absent, and T cells and B cells exhibited a scattered distribution (**Figures 2A, B**). Conversely, the LSG of pSS patients showed a significant presence of T cells, especially CD4^+^T cells, and B cells (**Figures 2C, D**). GC were accurately defined by HE staining combined with IHC staining of CD3, CD20, and CD21 serial sections (30). Serial sections of CD4, ICOS, and CXCR5 revealed that Tfh cells were predominantly distributed in the GC area (**Figure 2C**). However, only a small number of Tfh cells were observed in single smaller foci lesions lacking GC-like structures (**Figure 2E**), while more Tfh cells were distributed in patients with large lesions (**Figure 2F**). By performing RNA-sequencing on the LSG samples, we further examined the expression of T cell subsets and found a significant increase in the expression of key Tfh cell genes in a subset of pSS patients (**Figure 2G**). Statistical analysis found that the expression of these genes was significantly higher in the pSS group (**Figure 2H**). These findings indicate that Tfh cells were primarily distributed in a subset of LSGs of pSS patients exhibiting large lesions or GC-like structures.

### Inflammatory response-related genes are enriched in the Tfh-high cluster

To further investigate the role of Tfh cells in pSS, patients were categorized into a Tfh-low group and a Tfh-high group based on the median number of Tfh cells. Compared to the Tfh-low group, the Tfh-high group exhibited a higher positive rate of sicca syndrome, ANA, rheumatoid factor (RF), and hypergammaglobulinaemia, as well as elevated levels of serum IgG (**Table 2**; **Supplementary Table S4**). It is worth noting that increased circulating Tfh cells have been associated with elevated serum IgG levels and decreased complement C4 (19).

**Table 2:** Clinical information for the 82 patients used for RNA sequencing grouped according to Tfh cell infiltration.

Clinical characteristic	Tfh-high (n=34)	Tfh-low (n=34)
*Gender (Female)*	29/34	25/34
*Age*	49.18 ± 13.26	49.68 ± 15.51
*Xerostomia (%)*	85.3**	52.9
*Xerophthalmia (%)*	61.8*	35.3
*ANA+ (%)*	94.1*	76.5
*RF (%)*	44.1**	8.8
*Anti-SSA+ (%)*	79.4	55.9
*Anti-Ro52+(%)*	58.8	52.9
*Anti-SSB+ (%)*	26.5	20.6
*IgG (g/L)*	17.43 ± 6.00**	13.30 ± 4.25
*IgA (g/L)*	3.01 ± 1.34	2.66 ± 1.08
*IgM (g/L)*	1.64 ± 1.04	1.41 ± 0.80
*C3 (g/L)*	1.08 ± 0.16	1.15 ± 0.22
*C4 (g/L)*	0.27 ± 0.09	0.32 ± 0.10
*Hypergammaglobulinaemia (%)*	44.1**	14.7
*Hypocomplementemia (%)*	2.9	2.9

Data are mean ± standard deviation (SD) or n (%). *p<0.05; **p<0.01; ***p<0.001. ANA= anti-nucleic antibody. RF, rheumatoid factors.

**Figure 3:**
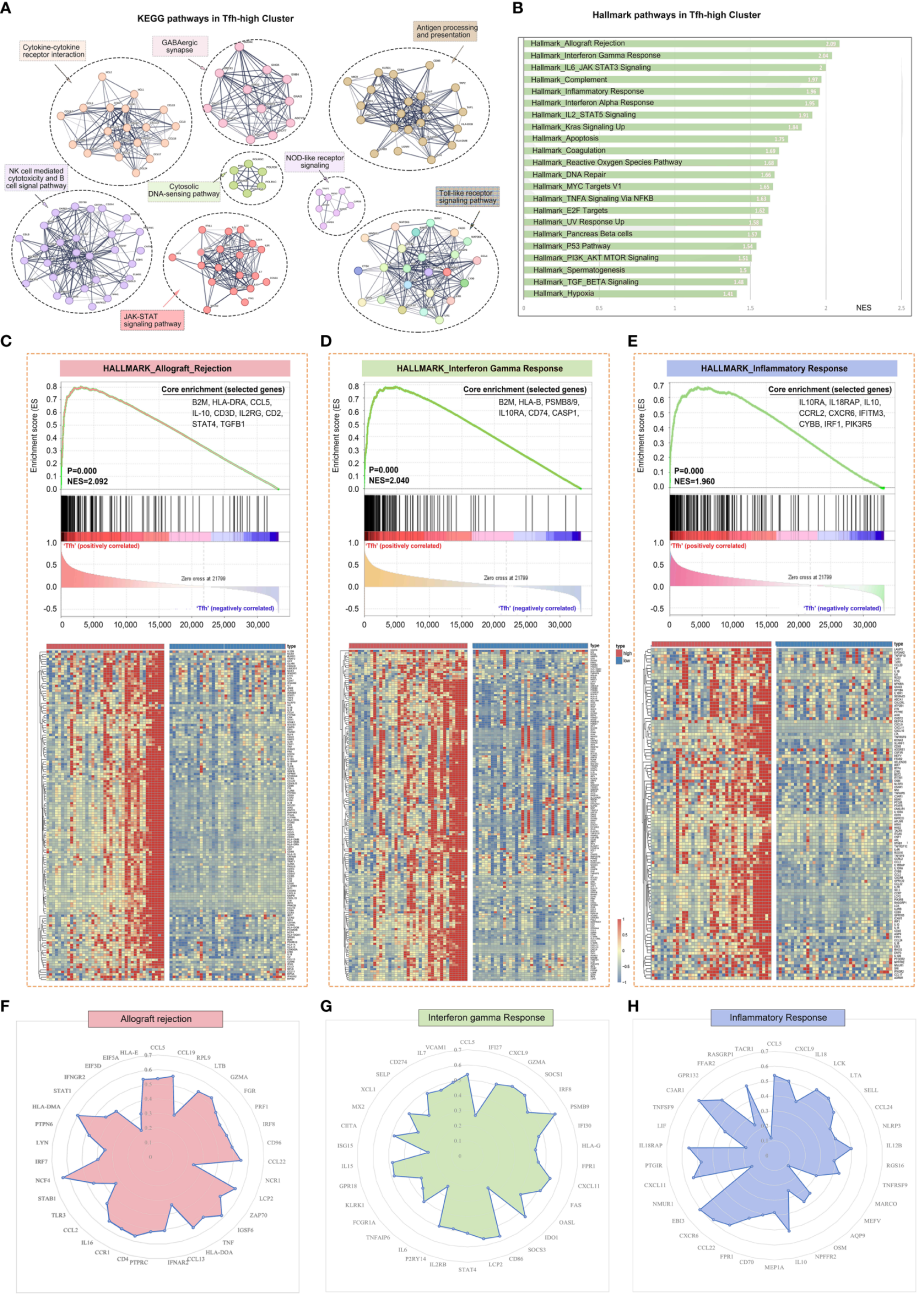
Gene expression patterns of different Tfh cell infiltration. The RNA sequencing patients were divided into Tfh-high and Tfh-low groups according to the expression of Tfh cell marker genes. **(A)** PPI network and Multi-KEGG pathway enrichment in Tfh-high group using STRING. **(B)** 22 significantly enriched signalling pathways obtained by Hallmark in Tfh-high group. **(C–E)** Gene Set Enrichment Analysis (GSEA) of Hallmark pathway enrichment for allograft rejection **(C)**, interferon-gamma response **(D)** and inflammatory response **(E)** in Tfh-high cluster. The heat maps at the bottom of the figures represent the gene expression of each signalling pathway. Some genes from allograft rejection **(F)**, interferon-gamma response **(G)** and inflammatory response **(H)** signalling pathways were randomly selected for correlation analysis with Tfh cell marker genes and displayed as radar maps.

By conducting KEGG pathway enrichment analysis using GSEA, we observed significant enrichment of the pathways in the Tfh-high group. These pathways included cytokine-cytokine receptor interaction, natural killer cell-mediated cytotoxicity, B cell receptor signaling pathway, antigen processing and presentation, Toll-like receptor signaling, and JAK-STAT signaling pathway (**Figure 3A**). Mapping the Hallmark pathways revealed 21 enriched pathways in the Tfh-high group, such as allograft rejection, interferon (IFN) gamma response, complement, and inflammatory response (**Figure 3B**). Tfh cell-mediated allograft rejection has been linked to chronic graft-versus-host disease following organ transplantation (31). Additionally, IFN response and inflammatory response play crucial roles in the pathogenesis of pSS (32). Genes associated with these three major signaling pathways were significantly enriched in the Tfh-high cluster (**Figures 3C–E**). Then, we randomly selected some genes from these three signaling pathways for correlation analysis with Tfh cell marker genes, and found that these genes showed a significant positive association with Tfh cells (**Figures 3F–H**). Collectively, these findings indicate an association between Tfh cells and the inflammatory response in pSS.

### Tfh cells are involved in the development of pSS disease through the regulation of immune response and metabolic processes

**Figure 4:**
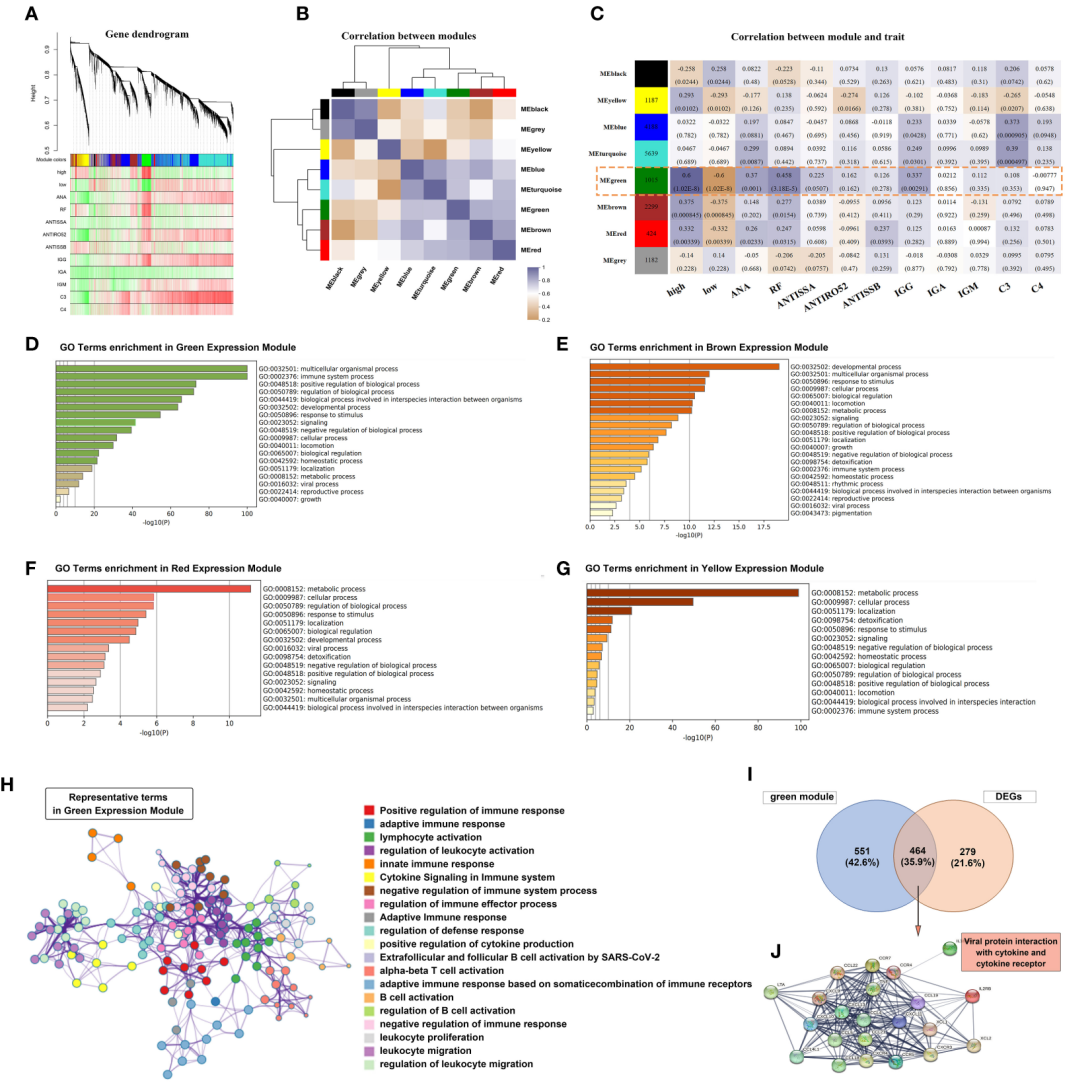
Tfh cell-related co-expression network generated using weighted gene co-expression network analysis (WGCNA) and gene enrichment analysis. **(A)** Different branches of the hierarchical clustering tree represented different gene co-expression modules (upper panel). The heat map represents the clinical indicators of the patients in each module. **(B)** A total of 8 modules were identified and the correlation was analyzed between the modules. The colour purple-grey represents a positive correlation and orange represents a negative correlation, with the shade of colour representing the level of correlation. **(C)** A heatmap chart showing module-trait relationships. The number in each module cell means the enriched genes number, and in each grid means the correlation coefficient and p-value. The correlation of variables was performed using the Pearson correlation coefficient. **(D–G)** The bar graph showed GO term enrichment analysis using Metascape in Green **(D)**, Brown **(E)**, Red **(F)** and Yellow **(G)** expression modules. **(H)** Significantly enriched clusters in the network marked with different colour balls, representing different GO terms, were annotated by Metascape. **(I)** Venn diagram depicted the 464 genes that overlapped with the DEGs and 1015 genes of the Green module. **(J)** Construction of the PPI network based on the 464 genes.

To further investigate the role of Tfh cells in pSS, we performed a transcriptome-wide weighted gene co-expression network analysis (WGCNA) and identified eight main co-expression modules based on the High- and Low- Tfh cells clusters (**Figure 4A**). The correlation between these eight modules is presented in **Figure 4B**. Notably, the green module, consisting of 1015 genes, exhibited a significant association with positive rates of ANA and RF, as well as elevated IgG levels (**Figure 4C**), which aligns with our clinical data. Metascape analysis based on the Gene Ontology (GO) terms of the green module revealed enrichment of gene groups related to the ‘multicellular organismal process’ and ‘immune system process’ (**Figure 4D**). We repeated the same analysis with the Brown, Red, and Yellow modules. The term ‘developmental process’ was predominant in the Brown module, while the term ‘metabolic process’ was predominant in both the Red and Yellow modules (**Figures 4E–G**). Furthermore, we constructed a hub term enrichment network based on the terms ‘multicellular organismal process’ and ‘immune system process’. As shown in **Figure 4H**, numerous immune system processes were located in the core of the network.

A total of 743 DEGs were identified between patients with pSS and non-pSS. Out of these, 464 genes intersected with the Green module (**Figure 4I**). We selected the most significantly different genes (| logFC | > 1.5) and constructed a PPI network. Notably, the most enriched category in the network was ‘Viral protein interaction with cytokine and cytokine receptor’ (**Figure 4J**), indicating a significant correlation between Tfh cell-mediated immune response and the IFN response following exposure to viral antigens.

**Figure 5:**
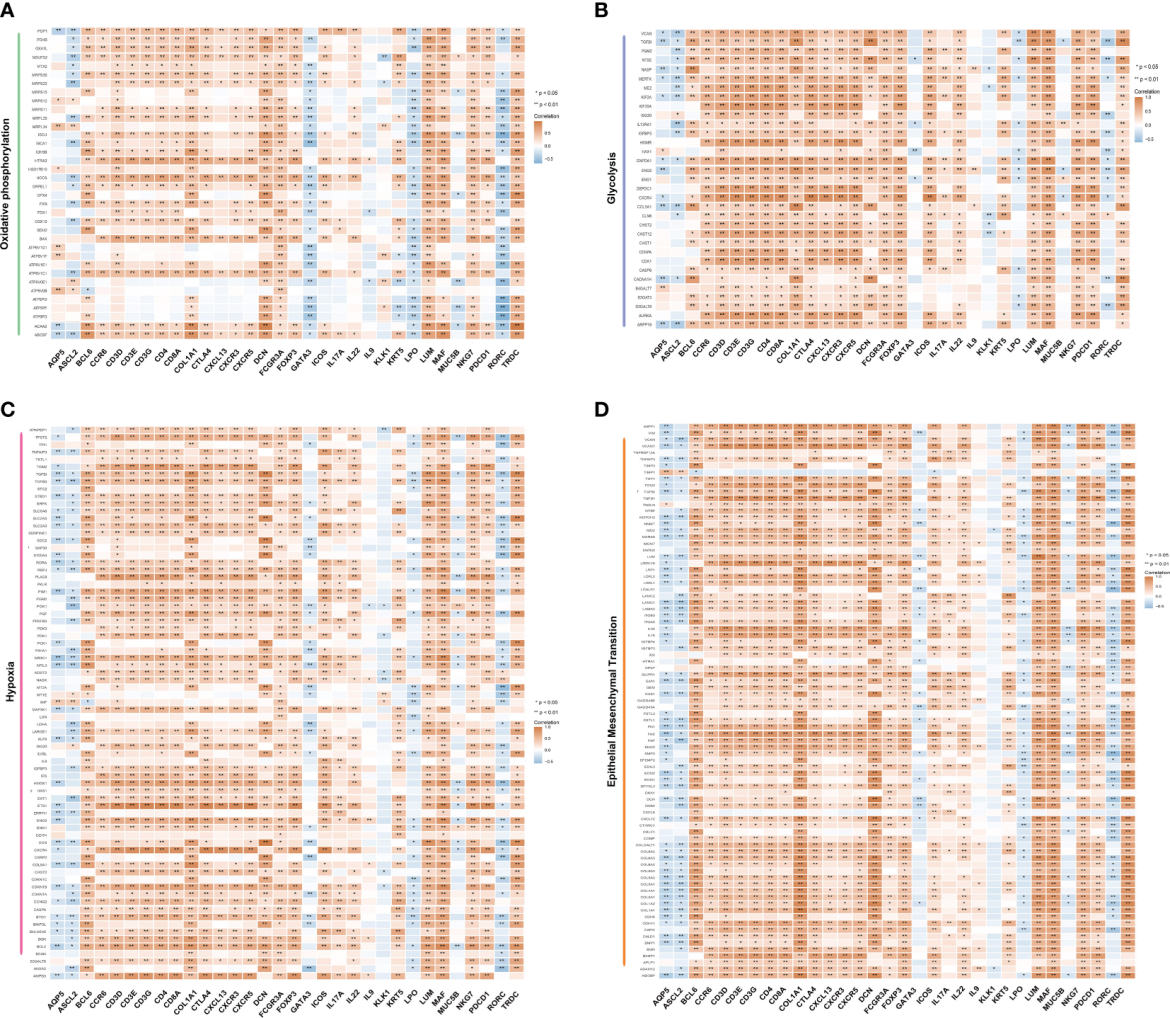
Correlation between metabolic pathways and T cells. Correlation between the marker genes of T cell subsets and the genes related to salivary gland function with oxidative phosphorylation **(A)**, glycolysis **(B)**, hypoxia **(C)** and epithelial-mesenchymal transition **(D)**, calculated using Spearman’s rank correlation analysis. The orange colour represents a positive correlation, and the blue colour represents a negative correlation (*p <0.05, **p <0.01). The shade of the colour represents the level of correlation.

According to our previous study (33), patients with pSS exhibited alterations in mitochondria-related genes associated with the mitochondrial metabolic pathway, oxidative phosphorylation, gluconeogenesis, TCA cycle, and pyruvate/ketone/lipid/amino acid metabolism. The interaction between salivary gland epithelial cells and lymphocytes (specifically, CD4^+^T cells and intraepithelial B cells) plays an important role in the pathological process of salivary glands (34). To explore this further, we performed a correlation analysis between marker genes of T cell subsets, genes related to salivary gland function and oxidative phosphorylation, glycolysis, hypoxia, and epithelial-mesenchymal transition. As shown in **Figure 5**, most key genes of T cell subsets as well as fibroblast marker genes (COL1A1, DCN, and LUM) showed a positive correlation with oxidative phosphorylation, glycolysis, hypoxia, and epithelial-mesenchymal transition. However, salivary gland function-related genes AQP5, LPO, KLK1, and MUC5B, and the Th2 cell marker gene GATA3 showed a significant negative correlation. Interestingly, ASCL2 and RORC, the key transcription factors of Tfh and Th17 cells, showed a similar negative correlation, although the marker genes of Tfh cells (BCL6, CXCR5, CXCL13, ICOS, and PDCD1) and Th17 cells (CCR6 and IL17A) showed a positive correlation. These findings provide valuable insights into the Tfh cell-mediated immune response and metabolic process.

### Characteristics of the immune microenvironment in LSG of different Tfh cell clusters in patients with pSS

**Figure 6:**
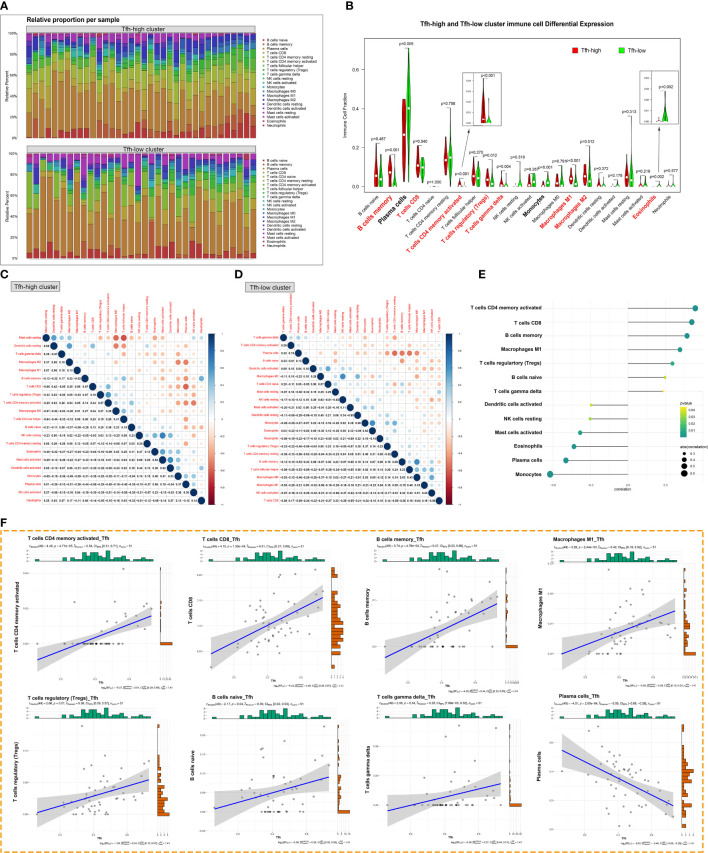
Tfh cell-associated glandular immune microenvironment. **(A)** The proportion of innate and adaptive immune cells infiltrating the glandular tissue was analyzed by the CIBERSORT algorism based on the degree of Tfh cells infiltration. **(B)** The violin diagram depicted the enriching level of innate and adaptive immune cells in the Tfh-high and Tfh-low clusters evaluated by the ssGSEA algorithm. Red bold font represents the groups of immune cells with significant differences. **(C, D)** Correlation analysis of infiltration between different immune cell populations in the Tfh-high **(C)** and Tfh-low **(D)** clusters. **(E)** Correlation analysis between Tfh cells and immune cell populations. **(F)** The scatter plot depicted the correlation between Tfh cells and representative immune cells.

We further analyzed the impact of Tfh cells on immune infiltration in patients with pSS. **Figure 6A** presents the proportion of immune cell infiltration in each sample of the Tfh-high and Tfh-low groups. Notably, the proportion of adaptive immune cells was significantly higher than that of innate immune cells in both groups, particularly in the Tfh-high group. The Tfh-high infiltration group showed a significant increase in adaptive immune cell numbers, including memory B cells, CD8^+^T cells, activated memory CD4^+^T cells, Tregs and γδ-T cells, and macrophages (**Figure 6B**). Plasma cells as well as some innate immune cells, such as monocytes and eosinophils, were more infiltrated in the Tfh-low group (**Figure 6B**). This finding may appear counterintuitive, considering that Tfh cells can assist B cell differentiation, maturation, and antibody production. We further examined the correlation between innate and adaptive immune cells in the two groups. In the Tfh-low group, the infiltration of Tfh cells showed a negative correlation with plasma cells. Whereas, in the Tfh-high group, the infiltration of Tfh cells showed a negative correlation with mast cells and DC and a positive correlation with memory B cells and macrophages (**Figures 6C, D**). Irrespective of the group, plasma cells showed a negative correlation with other immune cells (**Figures 6C, D**). It is apparent from **Figures 6E, F** that the infiltration of Tfh cells exhibited a positive correlation with activated memory CD4^+^T cells, CD8^+^ T cells, and memory B cells and a negative correlation with monocytes and plasma cells.

**Figure 7:**
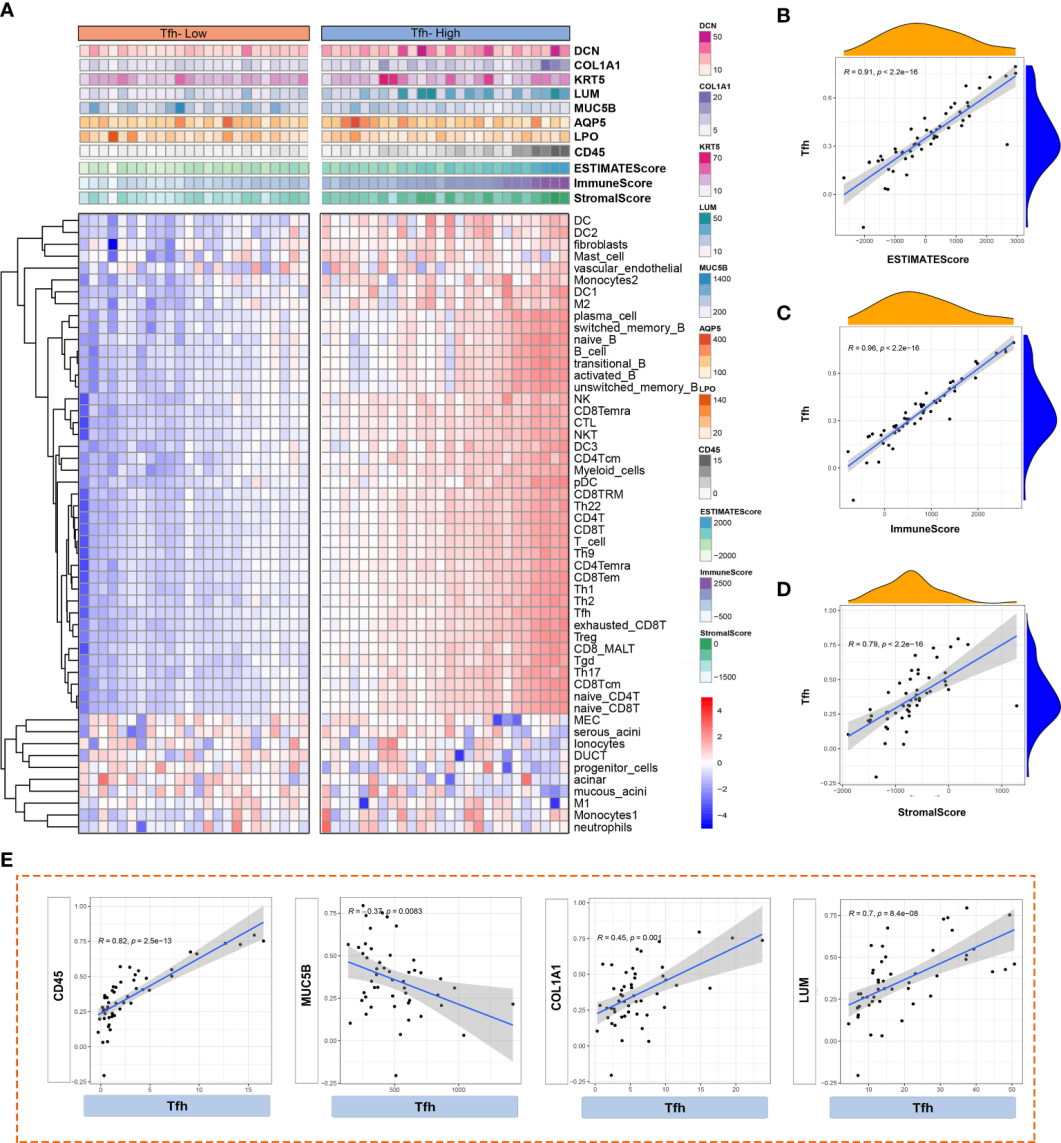
Analysis of immune cell infiltration based on different Tfh cell clusters. **(A)** Heatmap shows the enrichment scores of innate and adaptive immune cells and ESTIMATE in Tfh-high and Tfh-low clusters. Each column represents an individual patient sample. The colour red represents a higher expression and blue represents a lower expression. **(B–D)** The scatter plot depicted the correlation between Tfh cells and ESTIMATE score **(B)**, Immune score **(C)** and Stromal score **(D)**. **(E)** Scatter plot depicted the correlation between Tfh and interested glandular microenvironment-related genes (CD45, MUC5B, COL1A1 and LUM).

Abnormal activation of Tfh cells and B cells mediated by CXCL13 and IL21 has been observed in tumor tissues (35). Additionally, the presence of ectopic GC-like structures in LSGs is associated with an increased risk of lymphoma in patients with pSS (36). The ESTIMATE algorithm is commonly employed to assess overall immune infiltration and stromal content in tumor tissues (37). Leveraging Tfh cell infiltration, we comprehensively analyzed and illustrated immune infiltration in the glandular microenvironment along with the expression of LSG-related markers. As depicted in **Figure 7A**, the Tfh-high group exhibited predominant infiltration of innate and adaptive immune cells. Similarly, significantly higher Immune, Stromal, and ESTIMATE scores were observed in the Tfh-high group (**Figure 7A**), positively correlating with Tfh cells (**Figures 7B–D**). Furthermore, correlation analysis revealed a significant positive correlation between Tfh cells and CD45+ inflammatory cells, fibroblasts (COL1A1, LUM), whereas a negative correlation was observed with glandular function gene (MUC5B) (**Figure 7E**).

**Figure 8:**
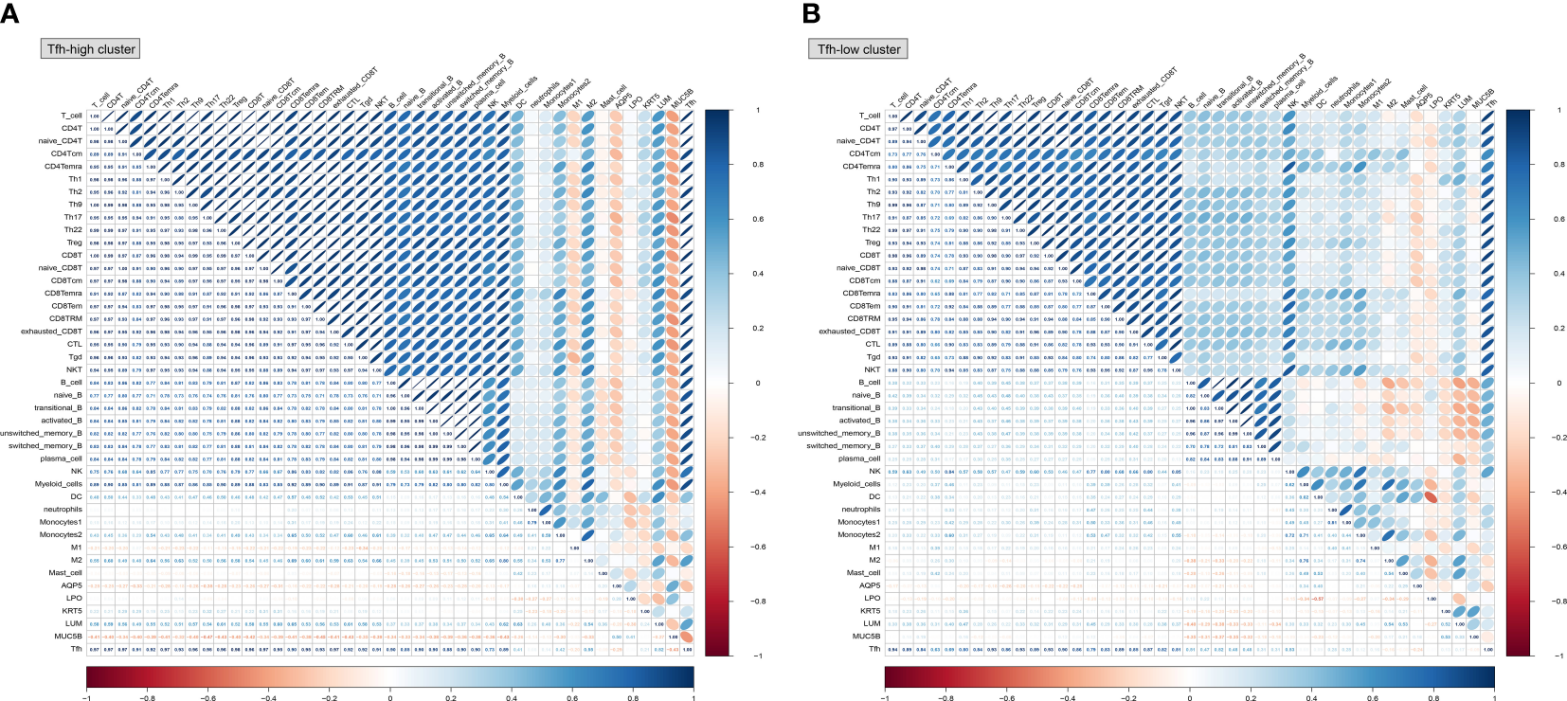
Correlation analysis between innate and adaptive immune cells based on different Tfh cell clusters. Based on the CellMarker 2.0 database and matching marker genes to known signature genes reported in previous studies, a total of 38 immune cell clusters and 5 genes related to salivary gland function were selected to perform the correlation analysis in the Tfh-high **(A)** and Tfh-low **(B)** clusters. The blue colour represents a positive correlation (Pearson’s r < 0) and the orange colour indicates a negative correlation (Pearson’s r > 0).

Further analysis based on single-sample Gene Set Enrichment Analysis (ssGSEA) explored the correlations between innate and adaptive immune cell populations, LSG marker genes, and Tfh cells. In the Tfh-high cluster (**Figure 8A**), Tfh cells exhibited a higher positive correlation with adaptive immune cells, macrophage 2 (M2), and fibroblasts (LUM). Notably, there was a consistent negative correlation between salivary gland function-related genes (AQP5 and MUC5B) and immune cells, regardless of whether they belonged to the Tfh-high or Tfh-low cluster, although the correlation was more pronounced in the Tfh-high cluster (**Figures 8A, B**). Collectively, these findings highlight the distinct immune cell infiltration microenvironments associated with varying degrees of Tfh cell infiltration.

## Discussion

Previous studies have emphasized the significance of Tfh cells, immune cell infiltration, and mitochondria-associated metabolic alterations (20, 33). In this study, we employed transcriptome sequencing technology to stratify pSS patients based on the level of Tfh cell infiltration due to its heterogeneous distribution. Notably, Tfh cell-related immune responses and altered metabolic processes were prominent features of the Tfh-high infiltration group. An intriguing finding was the high infiltration levels of Tfh cells showed a positive association with memory B cells and a negative association with plasma cells. The presence of ectopic GC in the biopsy of the LSG has been linked to an increased risk of lymphoma development in patients with pSS (17, 36). The mutual promotion of Tfh cells and B cells is an indispensable link to maintain GC formation (38). We hypothesize that plasma cells are present in the early stages of pSS, while the late stage is characterized by the infiltration of numerous Tfh cells accompanied by an abundance of memory B cells, which may serve as an important mechanism for GC formation and lymphoma occurrence.

In patients with pSS, there is an elevated proportion of CD4^+^CXCR5^+^Tfh cells in both salivary glands and peripheral blood (19, 39), Preliminary studies have indicated the importance of the IFN-α-JAK–STAT1 signaling pathway and the TOX-Bcl6 axis in Tfh cell activation (40). The dysregulation of the IL-21-TET2-AIM2-c-MAF pathway is a characteristic feature of lupus pathogenesis (41). In this study, the key transcription factors Bcl-6 shows an upward trend in the pSS group. Nevertheless, the high expression of PDCD1, CXCR5/CXCL13, and ICOS suggest that Tfh cells are terminally differentiated (38, 42). Targeting markers of terminally differentiated Tfh cells holds promise as an important approach to prevent pSS progression. Several drugs have been developed to target abnormal Tfh cell activation in the treatment of pSS, such as abatacept. By inhibiting co-stimulation and preventing Tfh cell activation in the treatment of pSS, such as abatacept. By inhibiting co-stimulation and preventing Tfh cell activation, abatacept has demonstrated improvement in disease activity, fatigue, and laboratory parameters in pSS patients, although its efficacy remains a subject of debate (43, 44). However, rituximab, targeting B cells (anti-CD20), has shown improved clinical outcomes in patients with pSS (45, 46). Additionally, anti-BAFF drugs, including belimumab (47) and ianalumab (48, 49), have been introduced in recent years to alleviate disease severity. However, a cure for pSS is still lacking, and further development of targets for the interaction between Tfh cells and B cells is necessary.

In the current stuty, the Tfh-high group exhibited a higher incidence of sicca syndrome, positive ANA, RF, and hypergammaglobulinaemia. A higher degree of immune cell infiltration or B-cell over-activation and a greater number of ectopic GCs were associated with higher titers of autoantibodies (e.g. ANA, RF, anti-Ro/SSA, anti-La/SSB) and a higher risk of MALT B-cell lymphoma in patients with pSS (1, 50). Localizing to B cell follicles and GCs, Tfh cells are defined by their ability to support GC formation, determine GC B cell differentiation into memory B cells and plasma cells to produce antibodies through cognate interactions (38). Tfh cells are significantly increased in the peripheral blood (51) and large lesions or GC-like structures in the LSG of pSS patients. Increased circulating Tfh cells and serum IL-21 are closely related to the increase of circulating IgG and the decrease of complement C4 (19, 52). Hence, we can infer that B cells interact with Tfh cells through GC to produce autoantibodies and affect the clinical manifestations of pSS patients.

The analysis revealed a notable finding related to the immune response mediated by Tfh cells. A crucial aspect of this immune response involves the virus protein-associated IFN response, which indicates the important role of the virus-IFN-B cell axis in the pathogenesis of pSS. Our results align with those of Bjork et al (53), who also observed increased immune activation and higher serum IgG levels following viral antigen exposure. Various viral agents have been identified as triggers for chronic immune system stimulation in pSS (54). Aberrant activation of the IFN system, particularly IFN-α, acts as a driver of pSS variability (55). IFN-α induces overexpression of the transcription factor TOX (56), promoting Tfh cell development in patients with pSS through the JAK–STAT1 signal pathway in pSS patients (40). Based on these data, it can be inferred that the IFN-α-JAK–STAT1-Tfh-B cell axis significantly contributes to the occurrence and progression of pSS.

Another important finding is the immune microenvironment alterations in the glands, leading to changes in local metabolic processes, which represent critical features of pSS pathology. The infiltration of immune cells creates a persistent hypoxic environment with heightened levels of glycolysis and oxidative phosphorylation. Abnormal immune metabolism has been observed in conditions such as rheumatoid arthritis (57) and systemic lupus erythematosus (58). Although we have identified overall metabolic changes in the immune microenvironment of LSGs in pSS patients, further investigation is needed to explore the metabolic alterations in specific innate and adaptive immune cells.

We acknowledge certain limitations in our research. Firstly, we did not fully validate the disease pattern axis associated with Tfh cells based on transcriptomics. Secondly, we have yet to demonstrate the immunomodulatory role of individual immune cells and their corresponding metabolic alterations. Additional studies are warranted to establish the relevance of Tfh cell-mediated pathogenesis in patients. Specifically, validation of immune responses and metabolic differences among specific immune cells is required.

## Conclusion

The present study aimed to investigate the impact of Tfh cells on patients with pSS. Firstly, we examined the distribution of Tfh cells in the pSS patient group and observed distinct stratification. In accordance with the distribution of Tfh cells, we noted that the Tfh-high group exhibited a higher prevalence of sicca syndrome, ANA, RF, and hypergammaglobulinaemia. We found an abnormal immune response primarily associated with virus-mediated IFN response and metabolic alterations characterized by a hypoxic glandular microenvironment and increased glycolysis and oxidative phosphorylation levels. Secondly, varying levels of Tfh cell infiltration were accompanied by different immune cell microenvironment states. Specifically, increased infiltration of Tfh cells correlated with reduced infiltration of plasma cells and increased infiltration of memory B cells. Through transcriptomics analysis, we provided evidence that Tfh cells contribute to the disease progression of pSS, laying the groundwork for further investigations into the underlying mechanisms of Tfh cells in pSS.

## Data availability statement

The datasets presented in this study can be found in online repositories. The names of the repository/repositories and accession number(s) can be found here: OER395404 (https://www.biosino.org/node/run/detail/OER395404).

## Ethics statement

The studies involving humans were approved by Ethics Committee of Ruijin Hospital, Shanghai Jiao Tong University School of Medicine. The studies were conducted in accordance with the local legislation and institutional requirements. participants provided their written informed consent to participate in this study.

## Author contributions

LJ, YX, NL and HX designed the overall research strategy. LJ and DL wrote the manuscript. DL, YYang and YG analyzed the data. LL, YYe, JH, YZ and JZ performed the experiments. All authors contributed to the article and approved the submitted version.
