# Correction: Unraveling the transcriptome-based network of Tfh cells in primary Sjogren syndrome: insights from a systems biology approach

**DOI:** 10.3389/fimmu.2026.1905826

**Published:** 2026-07-13

**Authors:** Danyang Luo, Lei Li, Yi Yang, Yulin Ye, Jiawei Hu, Yuan Zong, Jiawen Zhao, Yiming Gao, Haimin Xu, Ning Li, Yinyin Xie, Liting Jiang

**Affiliations:** 1 Department of Stomatology, Ruijin Hospital, Shanghai Jiao Tong University School of Medicine, Shanghai, China; 2 College of Stomatology, Shanghai Jiao Tong University, Shanghai, China; 3 Department of Pathology, Ruijin Hospital, Shanghai Jiao Tong University School of Medicine, Shanghai, China; 4 Shanghai Institute of Hematology, State Key Laboratory of Medical Genomics, National Research Center for Translational Medicine at Shanghai, Shanghai, China; 5 Ruijin Hospital, Shanghai Jiao Tong University School of Medicine, Shanghai, China

**Keywords:** primary Sjogren’s syndrome, labial salivary gland, T follicular helper cell, transcriptome sequencing, interferon

There was a mistake in **Table 1** and **Table 2** as published. Due to copy-paste errors during data integration, several records were duplicated and some correct data was missing. The corrected **Table 1** and **Table 2** appears below.

**Table 1:** Phenotypes of pSS and non-pSS patients compared according to 2016 ACR/EULAR diagnostic criteria.

Clinical characteristic	pSS (n=106)	Non-pSS (n=46)
*Gender (Female)*	93/106	37/46
*Age*	48.50 ± 12.19	52.48 ± 12.23
*ANA+ (%)*	91.5**	71.7
*Anti-SSA+ (%)*	87.7***	26.1
*Anti-Ro52+(%)*	72.6**	45.7
*Anti-SSB+ (%)*	34.0***	6.5
*IgG (g/L)*	18.21 ± 9.10***	12.74 ± 3.79
*IgA (g/L)*	2.90 ± 1.30	2.68 ± 1.03
*IgM (g/L)*	1.50 ± 0.88	1.47 ± 0.82
*C3 (g/L)*	1.11 ± 0.23	1.13 ± 0.19
*C4 (g/L)*	0.27 ± 0.09	0.31 ± 0.12
*Hypergammaglobulinaemia (%)*	28.3**	10.9
*Hypocomplementemia (%)*	2.8	6.5

Data are mean ± standard deviation (SD) or n (%). *p<0.05; **p<0.01; ***p<0.001.

ANA, anti-nucleic antibody.

**Table 2:** Clinical information for the 82 patients used for RNA sequencing grouped according to Tfh cell infiltration.

Clinical characteristic	Tfh-high (n=34)	Tfh-low (n=34)
*Gender (Female)*	29/34	25/34
*Age*	49.18 ± 13.26	49.68 ± 15.51
*Xerostomia (%)*	85.3**	52.9
*Xerophthalmia (%)*	61.8*	35.3
*ANA+ (%)*	94.1*	76.5
*RF (%)*	44.1**	8.8
*Anti-SSA+ (%)*	79.4	55.9
*Anti-Ro52+(%)*	58.8	52.9
*Anti-SSB+ (%)*	26.5	20.6
*IgG (g/L)*	17.43 ± 6.00**	13.30 ± 4.25
*IgA (g/L)*	3.01 ± 1.34	2.66 ± 1.08
*IgM (g/L)*	1.64 ± 1.04	1.41 ± 0.80
*C3 (g/L)*	1.08 ± 0.16	1.15 ± 0.22
*C4 (g/L)*	0.27 ± 0.09	0.32 ± 0.10
*Hypergammaglobulinaemia (%)*	44.1**	14.7
*Hypocomplementemia (%)*	2.9	2.9

Data are mean ± standard deviation (SD) or n (%). *p<0.05; **p<0.01; ***p<0.001. ANA= anti-nucleic antibody. RF, rheumatoid factors.

File **Supplementary Table 1** and **Supplementary Table 4** were erroneously published with the original version of this paper. The files have now been replaced.

The original version of this article has been updated.

