# Supplementary material for: Unraveling the transcriptome-based network of tfh cells in primary sjogren syndrome: insights from a systems biology approach
Source: Front Immunol. 2023 Aug 10;14:1216379. doi: 10.3389/fimmu.2023.1216379 (PMC10448518; doi:10.3389/fimmu.2023.1216379)
Supplement: Supplementary file 5 [file Table1.docx]

**SUPPLEMENTARY TABLE**

Table S1 Primary antibodies detailed information

| antigen | dilution/concentration | Cat. # | | Company and Nation | |
| --- | --- | --- | --- | --- | --- |
| ***Immunohistochemical staining*** | | | | |  |
| CD3 | undiluted | GA503 | Dako, Denmark | |  |
| CD4 | undiluted | IR649 | Dako, Denmark | |  |
| CD20 | undiluted | GA604 | Dako, Denmark | |  |
| CD21 | 1:500 | ab75985 | Abcam, UK | |  |
| ICOS | 1:500 | ab224644 | Abcam, UK | |  |
| CXCR5 | 1:200 | ab254415 | Abcam, UK | |  |
| ***Immunofluorescence staining*** | | | | |  |
| CD4 | 1:100 | ab133616 | Abcam, UK | |  |
| CXCR5 | 1:100 | ab254415 | Abcam, UK | |  |
